# Differential Responses of Vanilla Accessions to Root Rot and Colonization by *Fusarium oxysporum* f. sp. *radicis-vanillae*

**DOI:** 10.3389/fpls.2015.01125

**Published:** 2015-12-18

**Authors:** Sayuj Koyyappurath, Geneviève Conéjéro, Jean Bernard Dijoux, Fabienne Lapeyre-Montès, Katia Jade, Frédéric Chiroleu, Frédéric Gatineau, Jean Luc Verdeil, Pascale Besse, Michel Grisoni

**Affiliations:** ^1^UMR C53, PVBMT, CIRAD, 3P, Saint-PierreLa Réunion, France; ^2^UMR 5004 B&PMP (INRA, CNRS, Supagro, UM), PHIVMontpellier, France; ^3^UMR 1098, AGAP (CIRAD, INRA, Supagro), PHIVMontpellier, France; ^4^UMR C53, PVBMT, Université de La Réunion, Saint DenisLa Réunion, France

**Keywords:** disease resistance, genetic resources, lignin, multiphoton microscopy, phenolic compounds, plant histopathology, *Vanilla planifolia*

## Abstract

Root and stem rot (RSR) disease caused by *Fusarium oxysporum* f. sp. *radicis-vanillae* (*Forv*) is the most damaging disease of vanilla (*Vanilla planifolia* and *V.* × *tahitensis*, Orchidaceae). Breeding programs aimed at developing resistant vanilla varieties are hampered by the scarcity of sources of resistance to RSR and insufficient knowledge about the histopathology of *Forv*. In this work we have (i) identified new genetic resources resistant to RSR including *V. planifolia* inbreds and vanilla relatives, (ii) thoroughly described the colonization pattern of *Forv* into selected vanilla accessions, confirming its necrotic non-vascular behavior in roots, and (iii) evidenced the key role played by hypodermis, and particularly lignin deposition onto hypodermal cell walls, for resistance to *Forv* in two highly resistant vanilla accessions. Two hundred and fifty-four vanilla accessions were evaluated in the field under natural conditions of infection and in controlled conditions using *in vitro* plants root-dip inoculated by the highly pathogenic isolate Fo072. For the 26 accessions evaluated in both conditions, a high correlation was observed between field evaluation and *in vitro* assay. The root infection process and plant response of one susceptible and two resistant accessions challenged with Fo072 were studied using wide field and multiphoton microscopy. In susceptible *V. planifolia*, hyphae penetrated directly into the rhizodermis in the hairy root region then invaded the cortex through the passage cells where it induced plasmolysis, but never reached the vascular region. In the case of the resistant accessions, the penetration was stopped at the hypodermal layer. Anatomical and histochemical observations coupled with spectral analysis of the hypodermis suggested the role of lignin deposition in the resistance to *Forv*. The thickness of lignin constitutively deposited onto outer cell walls of hypodermis was highly correlated with the level of resistance for 21 accessions tested. The accumulation of p-coumaric and sinapic acids, two phenolic precursors of lignin, was observed in the resistant plants inoculated with Fo072, but not in the susceptible one. Altogether, our analyses enlightened the mechanisms at work in RSR resistant genotypes and should enhance the development of novel breeding strategies aimed at improving the genetic control of RSR of vanilla.

## Introduction

The genus *Vanilla* includes about 110 species of tropical climbing orchids. Among these, two species, *Vanilla planifolia* and *V. × tahitensis*, are cultivated for their aromatic compounds, particularly vanillin, present in high levels in their fruits which, after being cured, give commercial vanilla (Purseglove et al., 1981). Because vanilla vines have been only propagated clonally by cuttings, the genetic variability of cultivated plants is extremely narrow (Bory et al., 2008b; Lubinsky et al., 2008). This limited diversity hampers the capacity to select genotypes to adapt to abiotic or biotic constraints.

*Fusarium oxysporum* is a soil-borne fungus found worldwide. It is an anamorphic species that includes both pathogenic and non-pathogenic strains (Gordon and Martyn, 1997). The plant pathogenic forms infect their host by penetrating the roots, causing severe damage and yield losses on many economically important plant species (Michielse et al., 2009; Fourie et al., 2011). These are highly host-specific and are divided into 150 *formae speciales* based on the host they infect (Fourie et al., 2009; Bertoldo et al., 2015).

The root and stem rot (RSR) of vanilla is a serious disease caused by *F. oxysporum* f. sp. *radicis-vanillae* (*Forv*) in all vanilla producing countries (Tucker, 1927; Koyyappurath et al., 2015). The disease starts with the browning and death of underground roots, followed by the death of aerial roots. Subsequently the leaves and stem begins to shrivel and eventually the total collapse of the plant occurs. Control methods for RSR of vanilla reviewed by Tombe and Liew (2010) include good agronomic practices, the application of chemical fungicides or essential oils, or the use of biocontrol agents such as non-pathogenic strains of *F. oxysporum* and *Pseudomonas* and *Trichoderma* antagonists. However, none of these methods proved to be efficient enough to restore productivity of vanilla plots. Identifying and using genotypes resistant to *Fusarium* is considered to be the best alternative (Fravel et al., 2003).

Some degree of resistance is reported in *Vanilla* species such as *V. pompona*, *V. phaeantha*, *V. barbellata*, *V. aphylla*, and *V. andamanica* (Knudson, 1950; Theis and Jimenez, 1957; Divakaran et al., 2006) some of which have been used to create *V. planifolia* hybrids resistant to *Forv* (Theis and Jimenez, 1957; Delassus, 1963). Therefore identifying and breeding resistant genotypes is a promising strategy for controlling RSR of vanilla.

However, plant–pathogen interactions in vanilla are poorly described. Idioblast formation in response to *Forv* infection and differences in anatomy between genotypes have been previously reported in a vanilla hybrid (Theis and Jimenez, 1957; Tonnier, 1960; Stern and Judd, 1999), but understanding the colonization mechanism involved in the *Vanilla–Fusarium* pathosystem and how it changes according to plant genotype would allow specific breeding strategies.

In this study, we (i) evaluated the susceptibility of different vanilla accessions to RSR using a standardized screening method, (ii) developed an *in vitro* test of susceptibility to *Forv* enabling early selection of resistant accessions, (iii) described the colonization pattern of *Forv* in selected vanilla accessions, and (iv) investigated anatomy and kinetics of cellular events associated to *Forv* infection by using three accessions with different levels of resistance responses to the highly pathogenic *Forv* isolate Fo072. For the first time, *V. planifolia* accessions with high level of resistance to *Forv* have been identified which opens promising perspective for a better control of RSR by breeding.

## Materials and Methods

### Fungal Isolates and Vanilla Genotypes

The highly pathogenic isolate of *F. oxysporum* f.sp. *radicis-vanillae* Fo072 (Koyyappurath et al., 2015), was used in inoculation experiments. This isolate was selected because of its stability, high pathogenicity, and aggressiveness on susceptible vanilla accessions. Fo072 was grown on PDA plates and stored in darkness at 25 ± 2°C. Inoculum was prepared as described in Koyyappurath et al. (2015). In brief, the mycelia grown on PDA for 7 days were gently scraped to a minimal liquid media and incubated for 5 days at 25°C on a rotary shaker at 125 rpm. The mycelia were then removed by filtration to prepare a conidial suspension adjusted to 10^6^ conidia ml^-1^.

The *Fusarium oxysporum* isolate Fo072 used in this study was deposited under the accession number MIAE01765 in the fungal collection of microorganisms of agro-environmental interest (MIAE) at UMR Agroécologie, Institut National de la Recherche Agronomique, Dijon, France (http://www6.dijon.inra.fr/umragroecologie_eng/Plateformes/ERB/Microorganismes-du-Sol).

The vanilla materials for the study were provided by the VATEL Biological Resources Center (Roux-Cuvelier and Grisoni, 2010) which maintains different species, hybrids, and progenies of vanilla in Reunion Island. This material was previously characterized for taxonomy and genetic diversity (Bory et al., 2008a,b; Bouétard et al., 2010). A total of 254 vanilla accessions cultivated in shade houses or *in vitro* were selected for this study (**Table 1**). Field plants were cultivated under a 60% shade-net and supplemented twice a year with compost made of coconut husk, sugarcane bagasse and filter-cake. The *in vitro* plants were grown in basal Murashige and Skoog media (Duchefa Biochemie, Nederland) without any growth hormone.

**Table 1:** Number of *Vanilla* accessions evaluated for RSR resistance in the field in 2009 (RSR index) and 2013 (RSR level), and by *in vitro* inoculation test (AUDPC).

Type of material	Code	Section^1^	Field 2009	Field 2013	*In vitro* (AUDPC)	Global^2^
**Species**						
*Vanilla bahiana*	bah	Xanata	6	8	4	11
*V. chamissonis*	cha	Xanata	–	2	1	2
*V. cribbiana*	cri	Xanata	–	5	–	5
*V. imperialis*	imp	Xanata	–	2	–	2
*V. insignis*	ins	Xanata	–	1	1	1
*V. lindmaniana*	lin	Xanata	1	1	–	1
*V. odorata*	odo	Xanata	2	2	–	3
*V. planifolia*	pla	Xanata	90	109	79	174
*V. pompona*	pom	Xanata	19	25	1	25
*V. × tahitensis*	tah	Xanata	1	1	1	1
*Vanilla* sp.	sp.	Xanata	1	1	1	1
*V. africana*	afr	Tethya	–	2	–	2
*V. crenulata*	cre	Tethya	4	4	1	4
*V. humblotii*	hum	Tethya	–	2	1	2
*V. madagascariensis*	mad	Tethya	–	3	1	3
*V. phalaenopsis*	pha	Tethya	–	1	1	2
*V. roscheri*	ros	Tethya	–	–	1	1
*Vanilla* sp.	sp.	Tethya	–	1	1	2
**Hybrids**						
Hyb. pla × pom	hyb	Xanata	1	1	4	5
Hyb. ins × bah	hyb	Xanata	–	–	2	2
Hyb. pla × phae^3^	hyb	Xanata	2	2	1	2
Hyb. pom × plan	hyb	Xanata	–	–	1	1
Hyb. pla × tah	hyb	Xanata	1	1	–	1
Hyb.[(pla × pom) × pla] × [(pla × pom) × pla]	hyb	Xanata	–	–	1	1
**Total**			**128**	**174**	**103**	**254**

*^1^According to Soto Arenas and Cribb, 2010. ^2^Number of accessions tested by at least one of the three methods. ^3^phae = V. phaeantha.*

### Evaluation of RSR Resistance in Field Conditions

The field performance of vanilla accessions was assessed on three plants per accession, in shade houses naturally infected with *Forv*. All plants were older than 3 years. Two sets of observations were conducted, in December 2009 and February 2013. In the first set, 128 accessions of vanilla were rated using seven disease parameters (**Table 2**) linked to plant growth and symptoms (Tucker, 1927). The second set of ratings was done on 174 accessions, including 125 accessions in common with the first set, using an overall disease rating comprising three levels, as follows: Null: no RSR symptoms and good vigor, Moderate: intermediate vigor and moderate root proliferation and necrosis, Severe: plant severely affected by RSR showing vine decay and limited growth.

**Table 2:** Growth (PS, SN, PV) and disease (SD, NR DR, AR) variables used for rating *Forv* resistance in the field.

Code: descriptor	Level	Significance/range
PS: plant size	0	Dead
	1	Small (<0.3 m^2^)
	2	Medium (∼1 m^2^)
	3	big (>1 m^2^)
SN: number of stems loops	0	1–4
	1	5–9
	2	10–19
	3	20–49
	4	>50
SD: percentage of grooved stem and decaying leaves	0	>60%
	1	40–60%
	2	20–40%
	3	5–20%
	4	<5%
PV: vigor of the plant (size and number of new shoots)	0	Null
	1	Low
	2	Medium
	3	High
NR : Number of roots descending along the stalk	0	<3
	1	4–9
	2	10–19
	3	20–39
	4	>40
DR: proportion of dried roots among the descending roots.	0	Very few
	1	Less than half
	2	About half
	3	More than half
	4	Almost all
AR: number of aerial roots	0	<2
	1	2–4
	2	5–9
	3	>9

*A resistant or healthy plant is expected to show high growth levels and low disease levels.*

### *In vitro* Plant Inoculation and Assay

To infect the vanilla plantlets with *Forv*, the root-dip inoculation method (Koyyappurath et al., 2015) was used. The method consists of dipping the roots of *in vitro* grown plantlets in the conidial suspension (10^6^ conidia ml^-1^) of Fo072 for 5 min. The control plants were dipped in sterile distilled water. For susceptibility assessment, the inoculated plantlets were transferred to plastic plots containing sterile coco fiber and bagasse as substrate and incubated in a growth chamber at 24–26°C, 75–95% relative humidity, and PAR light density of 120 μmoles m^-2^ s^-1^ with a photoperiod of 12 h. Disease on plantlet was rated as described in Koyyappurath et al. (2015). The susceptibility of the accessions was derived from the area under the disease progress curve (AUDPC) values recorded every 2 days. For histo-pathological imaging, the inoculated plantlets were incubated in sterile tubes at 25°C for a 12-h photoperiod prior to tissue preparation. Experiments were generally done once, using four to eight plants per accession. For the reference accessions (pla0001, pla0020, and pom0018) or questionable accessions the experiments were repeated at least two times.

### Wide Field Microscopy

Conventional wide field microscopy (WFM) was used to study the anatomy of vanilla accessions and the colonization pattern of pathogenic fungi. The *Forv*-inoculated vanilla roots were examined at 0–9 days post-inoculation (dpi) in at least three independent experiments. Root tissues excised from control and inoculated plants were fixed for 48 h with 4% paraformaldehyde in 0.2 M phosphate buffer (pH 7.2). The samples were dehydrated through a graded alcohol series (50, 70, and 90°) and impregnated in methyl methacrylate, then each sample was embedded in polymethyl-acrylate LKB historesin and polymerization was performed at 37°C for 24 h. Three μm thin sections were obtained using a microtome (Leica, Germany) and were double stained with Schiff reagent and Naphthol Blue-Black stain (Schiff-NBB). The fixed slides were then viewed under a Leica DM6000 epifluorescence microscope (Leica Microsystems, Germany, objectives Leica 20x HC PL APO 0.7 and 40x) and images were acquired using Retiga 2000R camera (QImaging, Canada) and processed with Volocity software (Perkin-Elmer, UK).

For the anatomical studies of vanilla accessions, the roots from non-inoculated plants were collected and sectioned (90 μm) using a HM650 V vibrating blade vibratome (Thermo Scientific, Walldorf, Germany). The sections were then stained with 2% phloroglucinol (in ethanol) for 5 min. The sections were then transferred to a clean glass slide. A drop of 18% HCl was applied and a cover slip was mounted immediately. The slides were then observed under the Leica DM6000 epifluorescent microscope. Outer epidermal cell thickness, hypodermis thickness, and radial thickening of the hypodermal cells were measured at 10 different points of each root section using Image J 1.47v software (NIH, USA).

### Multi-photon Confocal Microscopic Analysis

The inoculated *in vitro* plants were examined for *Forv* infection and colonization at 2, 4, 7, and 9 dpi in at least two independent experiments. Infected roots were carefully taken out of the tubes and thin sections (90 μm) were obtained using a vibrating blade microtome as previously mentioned. These sections were placed in 10 mM phosphate buffer saline (PBS) and then stained with DAPI 300 nM (4′, 6-diamidino-2-phenylindole) for 5 min in the dark. DAPI, classical DNA dye, also stains polyphosphates and emits a yellow fluorescence. The stained cells were then washed twice in PBS. The sections were mounted on a glass slide and observed using a Zeiss LSM780 multiphoton microscope (Zeiss, Germany), equipped with a Chameleon Ultra II laser (Coherent, CA, USA). With the multiphoton microscope, the optimal excitation wavelength for DAPI is 720 nm and the filter blocks, with differential spectral properties, were set to those of DAPI (415–480 and 550–610 nm) and chlorophyll (660–700 nm). Image acquisition was performed using Zen software (Zeiss, Germany). The acquired image channels were merged and processed using Image J 1.47v software.

### Emissions Spectral Analysis

The multiphoton microscope with a Chameleon Ultra II tuneable laser (690–1080 nm range excitation, Coherent, Santa Clara, CA, USA) enables the excitation of secondary metabolites in a manner similar to a UV laser (Conéjéro et al., 2014; Talamond et al., 2015). Optimal excitation was obtained at a wave length λ = 720 nm and band-pass emission in the 410–650 nm range using an array of 32 photomultiplier tube (PMT) detectors (Zeiss), each with an 8.8 nm bandwidth.

This spectral detector yielded spectral images and emission spectra from the epidermal and hypodermal walls of fresh root sections of Fo072 inoculated or control vanilla accessions. After obtaining the spectral acquisitions, the Linear Unmixing (ZEN software, Zeiss, Jena, Germany) function was executed to separate, pixel by pixel, the mixed signals of six defined pure autofluorescent compounds namely ferulic acid, conyferylic acid, sinapinic acid, p-coumaric acid, caffeic acid, and quinic acid (Sigma-Aldrich, St. Quentin Fallavier, France), using the entire emission spectrum of each compound plus a residual channel. This image analysis showed each compound present in the sample with coded colors. In the residual channel, the intensity values represented the difference between the acquired spectral data and the fitted linear combination of the reference spectra.

### Data Analysis

All statistical analyses were performed with the R statistical software (R_Core_Team, 2015). The AUDPC was calculated using the *agricolae* R package (Mendiburu, 2014). Hierarchical classification of accessions based on their AUDPC values was done by recursive partitioning and complexity optimization using the *rpart* R package (Therneau et al., 2014). The field ratings dataset for plant development and symptom expression was analyzed with multiple correspondence analysis (MCA) using *ade4* R package (Chessel et al., 2004). The linear regression realized between the resistance level of the accessions, evaluated by the mean AUDPC values, and the hypodermal cell wall thickness and the hypodermal cell height, were tested for slope = 0 using the Wald test. The correlation between, average AUDPC and presence of radial lignification was tested using the Fischer exact test for count data in R. For all tests a 5% significance threshold was used.

## Results

### Field Evaluation of Accessions

**FIGURE 1:**
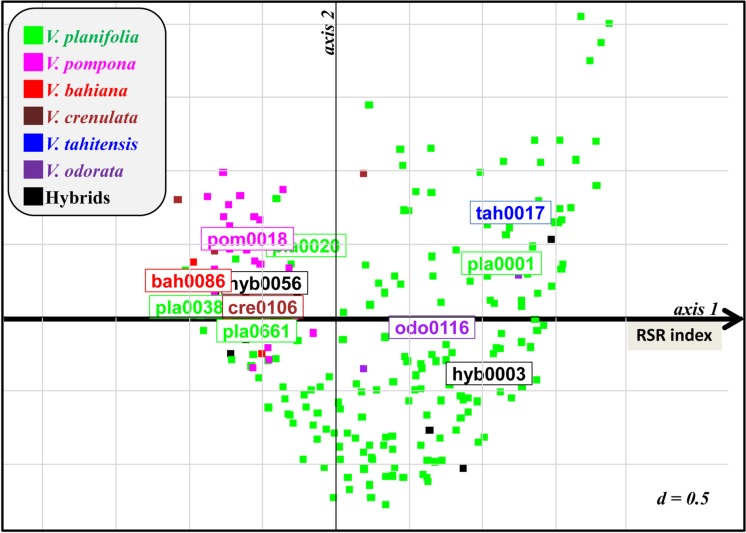
**First factorial plane of MCA for 128 vanilla accessions rated for RSR symptoms in the field in 2009 using seven variables (**Table 2** and Supplementary Figure S2).** Axis 1 segregates accessions according to their resistance level. Remarkable susceptible (on the right) and resistant (on the left) accessions are indicated. RSR index of accessions was defined as their coordinate on axis 1.

The vanilla vines cultivated in shade house collections during 3–9 years showed a range of performance regarding vegetative growth and RSR as exemplified in Supplementary Figure S1 However, for each accession, the development of the three replicated vines was identical. The presence of naturally occurring *Forv* in the shade houses was confirmed by fungus isolation and identification on selected plants showing RSR. This suggested that the variations in plant development could be related to differences in genetic susceptibility or resistance to *Forv*. In order to assess their level of resistance against natural inoculum, 177 accessions of at least 3 years of age were rated in field (shade houses) conditions. In the first set of observations, 128 accessions (three plants per accession) were evaluated for growth and symptoms using seven variables (**Table 2**). The first axis of MCA (**Figure 1** and Supplementary Figure S2) clearly segregated the plants according to vine development (PS, SN, SD, and PV) and proportion of dried roots (DR), the most affected plants being on the right side of the first factorial plane. The second axis correlated with total number of roots (NR). The 19 accessions of *V. pompona*, a species known to be resistant to RSR, and the six *V. bahiana* accessions, a sister species of *V. phaeantha*, another RSR-resistant species, were located on the extreme left of axis 1, as well as *V. sp.* CRV0068*, V. crenulata* and the two hybrids *V. planifolia* × *V. pompona* and *V. planifolia × V. phaeantha* (**Figure 1**). In contrast, accessions of *V. × tahitensis*, *V. odorata*, the hybrid *V. planifolia* × *V. × tahitensis*, along with most of the *V. planifolia* accessions were located on the right side of the first factorial plane, indicating their low level of resistance to *Forv*. A few *V. planifolia* accessions, such as pla0020, pla0038, or pla0240 were located on the left side, and showed development of vines similar to the resistant accessions. Since the six main MCA variables were highly correlated along the first axis, defining a RSR index, a global RSR rating of plant decay and disease symptoms, including three levels (null, moderate, and severe), was used in the subsequent evaluation of the accessions in the shade houses. The second set of ratings performed on 174 accessions was congruent with the first one for the 125 accessions that were rated twice in the field (Supplementary Figure S3): the majority of the plants having a low RSR index in 2009 were rated as “null” for RSR in 2013, and almost all plants having a high RSR index in 2009, rated “severe” in 2013.

### *In vitro* Germplasm Screening for Resistance to *Forv*

**FIGURE 2:**
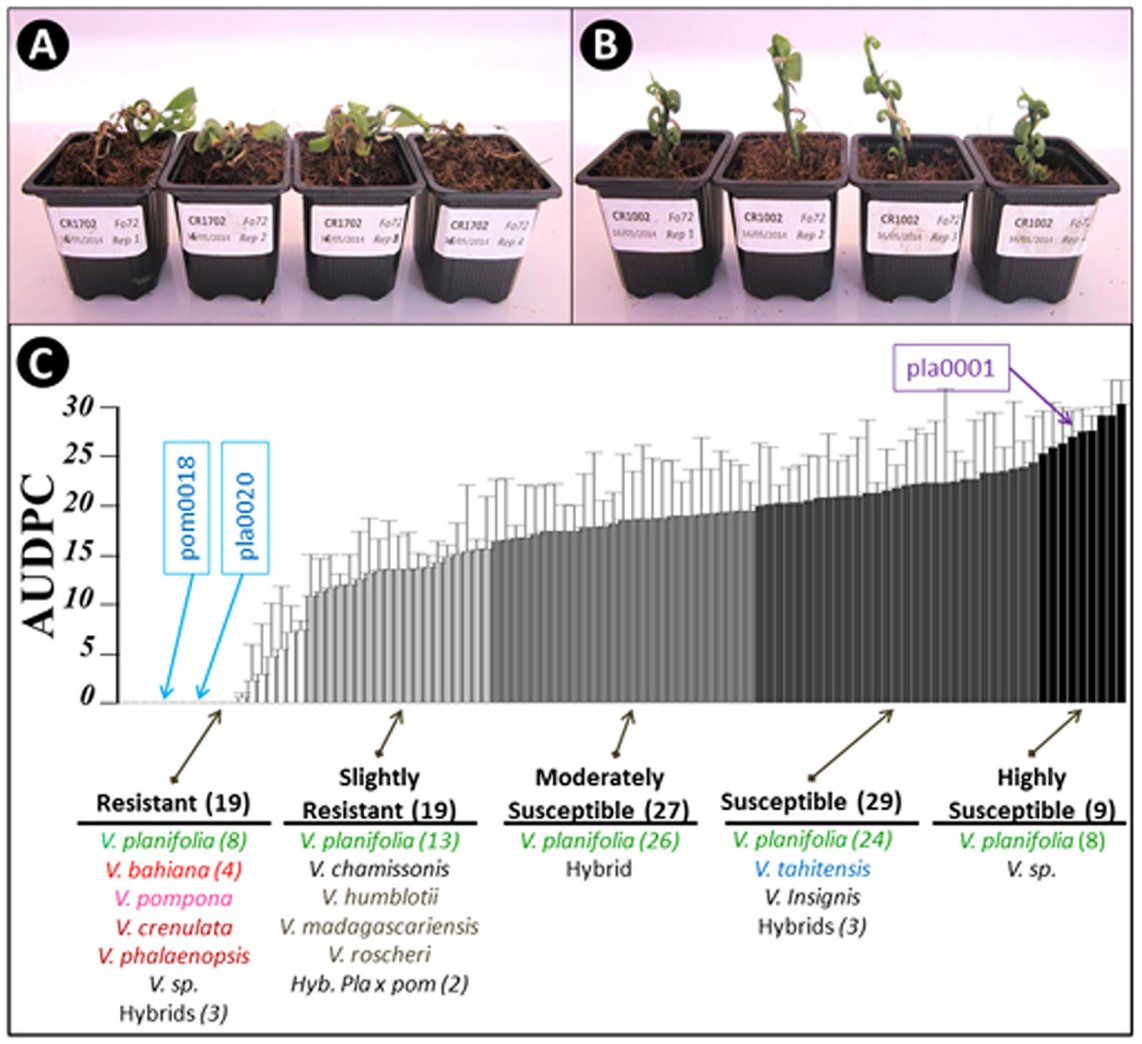
**Assessment of 103 *Vanilla* accessions for Fo72 resistance by *in vitro* plantlet assay. (A,B)** Appearance of susceptible and resistant genotypes, respectively, 15 dpi. **(C)** The 103 accessions were split into five classes according to average AUDPC values (4 plants per accession). The whiskers indicate the upper bound of asymptotic 95% confidence intervals of the average AUDPC values. The number in brackets indicates the number of accessions in each group or species, when greater than 1. The position of the three accessions selected for the histopathological studies (pla0001, pla0020, and pom0018) are highlighted.

One hundred and three accessions of vanilla (79 *V. planifolia*, 15 accessions from 11 *Vanilla* species and nine interspecific hybrids) were inoculated with Fo072 using the root-dip inoculation method. The disease symptoms started to appear on the susceptible accessions on the 4th dpi. Disease severity ratings were continued until 15 dpi, when most of the susceptible accessions reached total collapse (**Figures 2A,B**). No symptoms were observed on mock-inoculated plants. The accessions had a wide range of survival rates against the fungus with average AUDPC values ranging from 0 to 29.4 (**Figure 2C**). The hierarchical clustering analysis split the accessions into five classes based on the average AUDPC values, namely highly susceptible (HS), susceptible (S), moderately susceptible (MS), slightly resistant (SR), and resistant (R). Among the 103 accessions tested, 19 occupied the R class with weak or no symptom and an AUDPC average score lesser than 7. The plants in this class comprised eight *V. planifolia* accessions, four *V. bahiana* accessions, four other species (*V. pompona, V. crenulata, V. phalaenopsis*, and *V.* sp. CR0068) and three interspecific hybrids including *V. pompona* or *V. phaeantha* as a parent. To our knowledge, this is the first report of *V. planifolia* resistant to *Forv*. Interestingly seven of the eight *V. planifolia* accessions showing a high level of resistance were seedlings obtained from selfed-progenies of ordinary *V. planifolia* (**Table 3**). The remaining resistant *V. planifolia* accession originated from a vanilla plot in Reunion Island. At the other extreme, the S and HS classes were occupied by 27 *V. planifolia* accessions, *V. × tahitensis, V. insignis, V.* sp., plus two hybrids. The average AUDPC scores ranged from 19 to 29.4. The intermediate classes (SR and MS) contained 46 accessions with an AUDPC average score ranging from 10.5 to 18.9. In this group there were 39 *V. planifolia* accession, *V. chamissonis*, three African leafless species (*V. madagascariensis, V. roscheri and V. humblotii*), one *V. insignis × V. bahiana* hybrid, and two *V. planifolia* × *V. pompona* hybrids.

**Table 3:** Resistance to Fo72 of 52 *V. planifolia* selfed-progenies obtained from different accessions of ordinary *V. planifolia* susceptible to *Forv*.

Resistance group^1^	Parent							
	pla0035	pla0040	pla0041	pla0051	pla0066	pla0136	ordinary	Total
R [0;7.1]		1	2		2	1	1	7 (13.5%)
SR [11.2;15.1]		5	2		1			8 (15.4%)
MS [15.9;18.9]		8	4	2	5			19 (36.5%)
S [19.5;23.6]	1	5	5		3			14 (26.9%)
HS [24.5;29.4]		1	2		1			4 (7.7%)
**Total**	**1**	**20**	**15**	**2**	**12**	**1**	**1**	**52 (100%)**

*^1^R, Resistant; SR, slightly resistant; MS, moderately susceptible; S, susceptible; HS, highly susceptible. The range of AUDPC values for each group is indicated between brackets.*

The high level of resistance of pom0018 and pla0001 and the susceptibility of tah017 and pla0038 to *Forv* was not specific to the Fo72 isolate since replicated experiments conducted *in vitro* using different isolates, four highly and five moderately pathogenic isolates (Koyyappurath et al., 2015), gave similar results (data not shown).

### Correlations between Field and *In vitro* Evaluations of RSR Resistance

**FIGURE 3:**
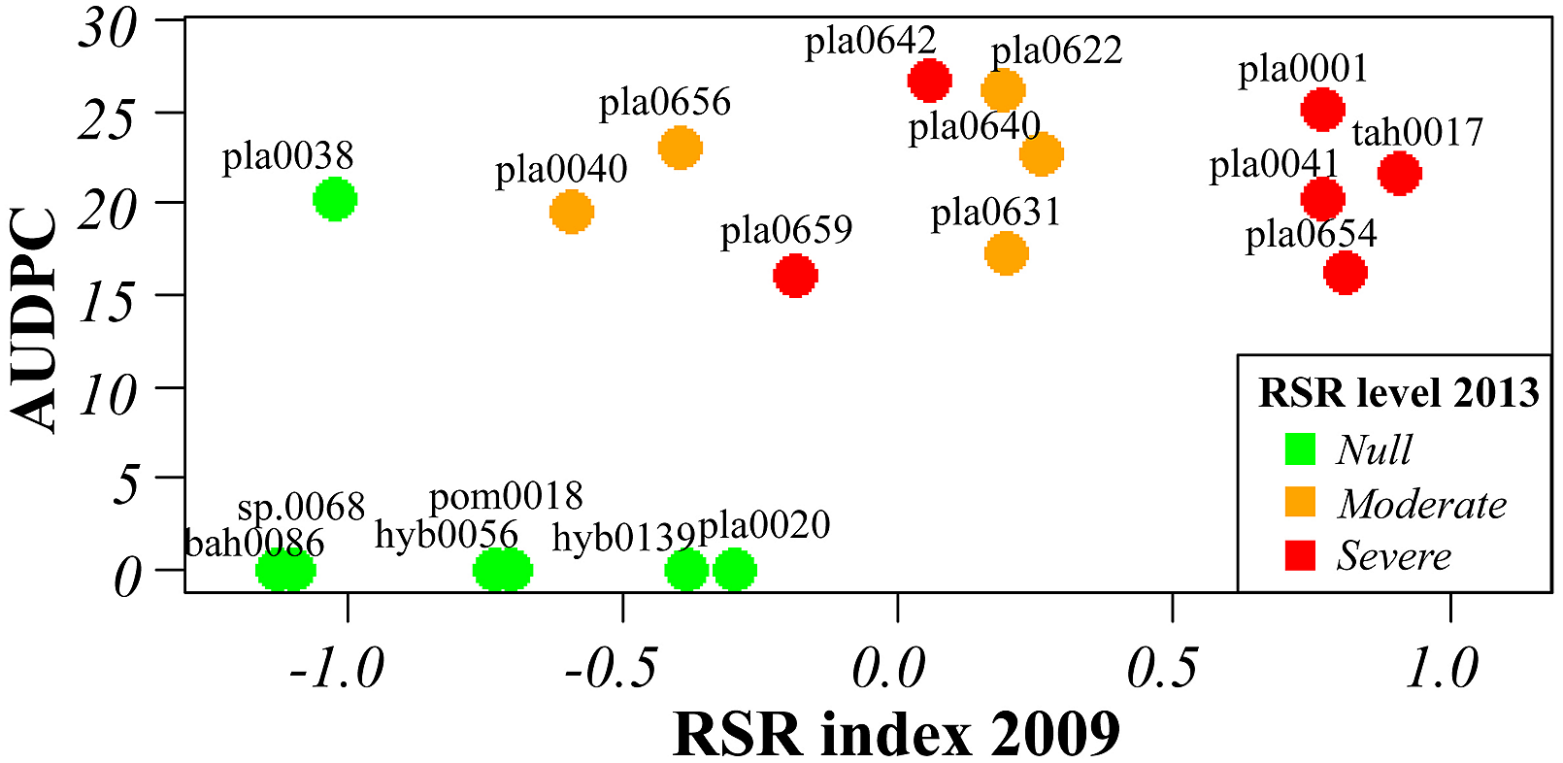
**Correlation between RSR symptoms in the field rated in 2009 and resistance to *Forv* estimated by AUDPC value of *in vitro* plantlets inoculated with Fo072.** Color of dots indicates the resistance level rated in the field in 2013 (cf. Supplementary Table S1).

Several of the accessions evaluated by AUDPC after root inoculation *in vitro* were also assessed for growth and symptoms after cultivation for 3–9 years in agricultural environment. A good correlation was observed between AUDPC values and the disease index in the field for the 19 and 26 accessions evaluated in 2009 and in 2013, respectively (**Figure 3** and Supplementary Table S1). All the accessions exhibiting resistance *in vitro* were resistant in the field and most accessions that showed moderate to severe RSR in the field were classified as very to MS in *in vitro* tests. However, a discrepancy was observed for two accessions that performed well in the field but were susceptible to Fo072 in repeated *in vitro* tests (pla0038 an ins0087), and for two accessions that showed moderate symptoms in the field despite being classified as moderately resistant in *in vitro* tests (hum0108 and cha0666).

As a whole, our results confirmed the high variability of the interaction between *Vanilla* genotypes and *F. oxysporum*. It documented the varying levels of resistance and susceptibility to *Forv* within *Vanilla* genetic resources, and we reported, for the first time, the existence of *V. planifolia* accessions with high level of resistance to RSR.

Three accessions were selected from the two extreme classes of resistance for further histological studies: the HS *V. planifolia* pla0001, and the two resistant *V. planifolia* pla0020 and *V. pompona* pom0018 accessions.

### Colonization of the Vanilla Root Surface

After 2 days of incubation, the growth of Fo072 hyphae became visible on the surface of roots and had expanded to the whole plant by the 8th dpi. On the susceptible accession (pla0001) the hyphal network was more prominent and induced severe rot (Supplementary Figure S4) while hyphal development was generally less on resistant accessions.

### Colonization into the Vanilla Root Tissues

**FIGURE 4:**
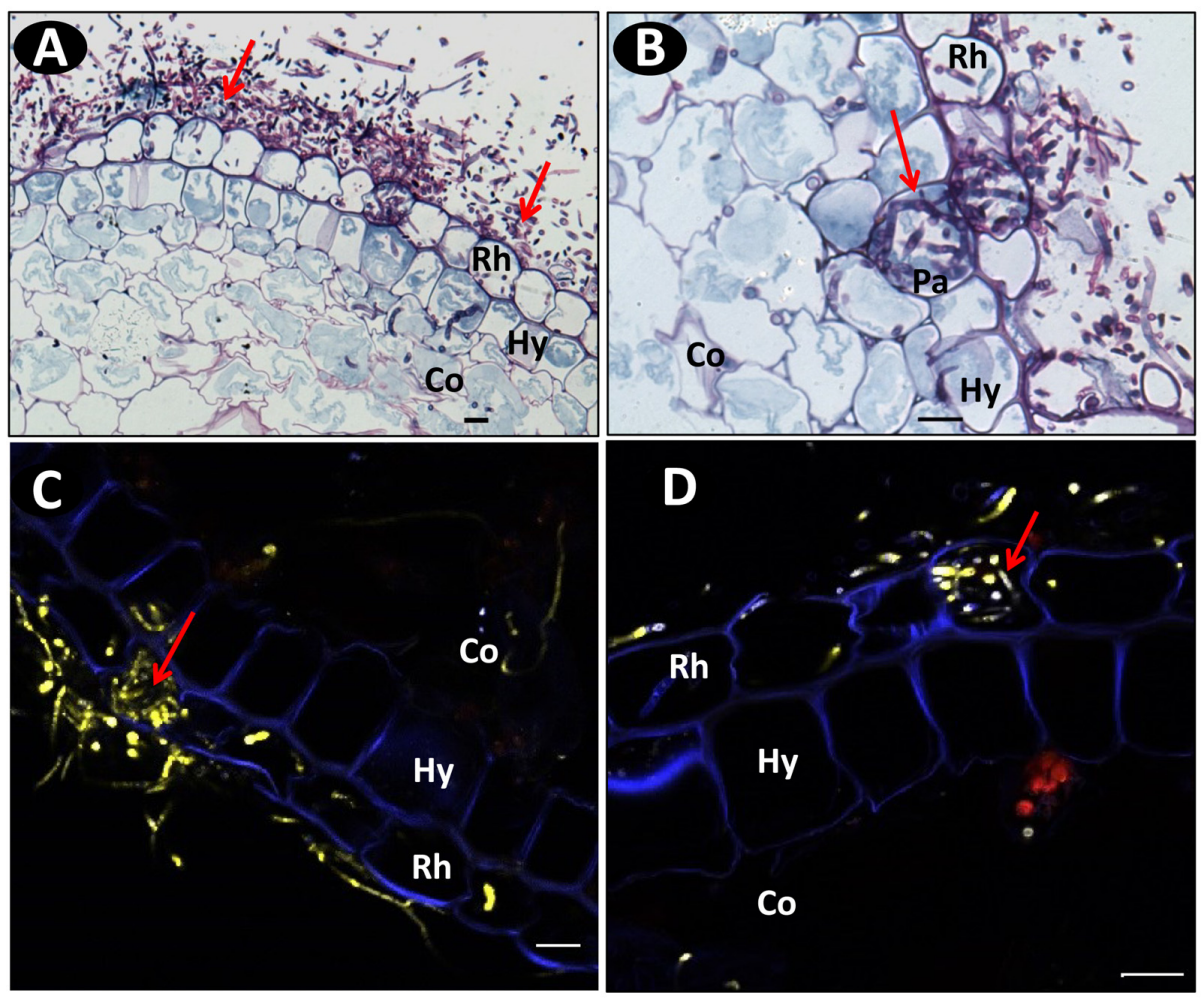
**Microscopic images of transverse root sections of pla0001 infected with Fo072 showing the colonization and penetration processes. (A)** wide field microscopy (WFM) images showing the abundant mycelia network (pointed with arrows) on pla0001 double-stained with Schiff reagent and Naphthol Blue-black stained at 3 dpi and **(B)** showing the penetration of Fo072 hyphae through the passage cells (Pa) of the hypodermal layer (Hy) in pla0001(denoted with arrows) at 3 dpi. **(C,D)** Multiphoton microscopic images showing transverse sections of Fo072-infected pla0001 stained with DAPI showing the formation of hyphal coiled structures (denoted with arrows) in the rhizodermal cells (Rh) during the penetration process. (Co) = Cortical region. Scale bar: 20 μm.

**FIGURE 5:**
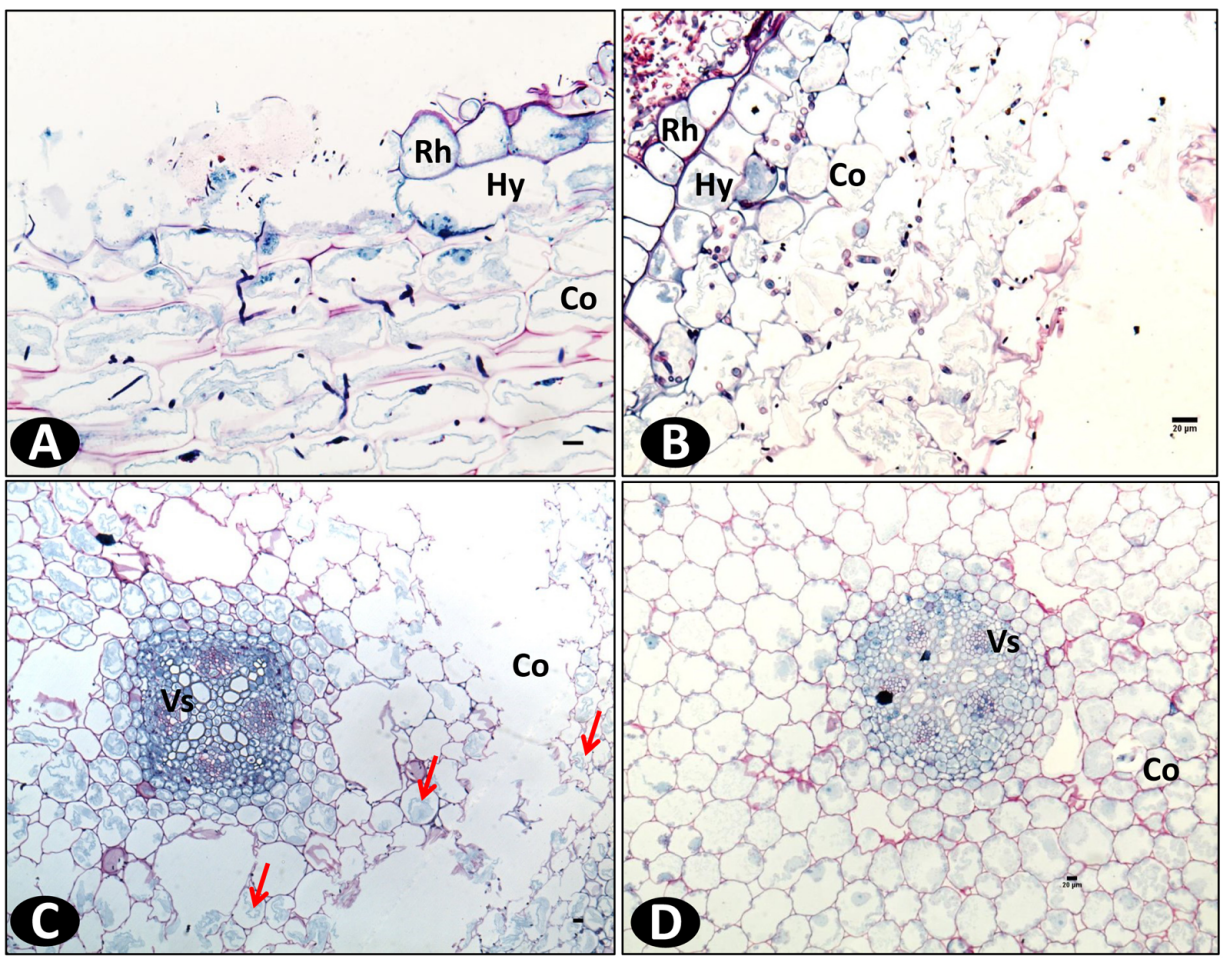
**Wide field microscopy images showing the colonization pattern of Fo072 in the *in vitro* roots of pla0001 double stained with Schiff reagent and Naphthol Blue-black at 7 dpi.** Longitudinal section **(A)** and transverse section **(B)** of pla0001 showing the intercellular colonization of hyphae in the cortical region. **(C)** represents the transverse section of pla0001 showing the plasmolysis of cytoplasm from the cell walls and total collapse of the cortical region (Co) with flaccid cells, thereby disturbing the root architecture. The colonization is affected mostly at the cortex region whereas the vascular (Vs) cells remains uninfected. **(D)** Displays the transverse section of non-inoculated pla0001 where the cortical cells remains firm and complete. Rh: Rhizodermis and Hy: hypodermis. Scale bar = 20 μm.

Schiff-NBB and DAPI-stained longitudinal and transversal root sections of inoculated plants were observed in WFM and under multiphoton microscopy, respectively. On the susceptible accession pla0001 the germination of conidia predominantly occurred at the hairy zone of the root at 2 dpi. The hyphae were attached to root hairs and epidermal cells. From 3 to 4 dpi, abundant mycelia networks had developed on the root surface mainly interwoven with the root hairs, and had begun to invade the epidermis (**Figure 4A**). The hyphae penetrated directly into the epidermal cells and formed a coiled structure (**Figures 4B–D**). Then they proceeded through the hypodermis specifically across passage cells and reached the cortex (**Figure 4B**). At 7 dpi, the hyphae invaded the cortex through the intercellular spaces (**Figures 5A,B**) and induced plasmolysis of the adjacent cortical cells (**Figures 5C,D**). This disruption of the cortical area concurred with the softening of the root tissue during infection. At 9 dpi no hyphae had reached the vascular cylinder (data not shown).

**FIGURE 6:**
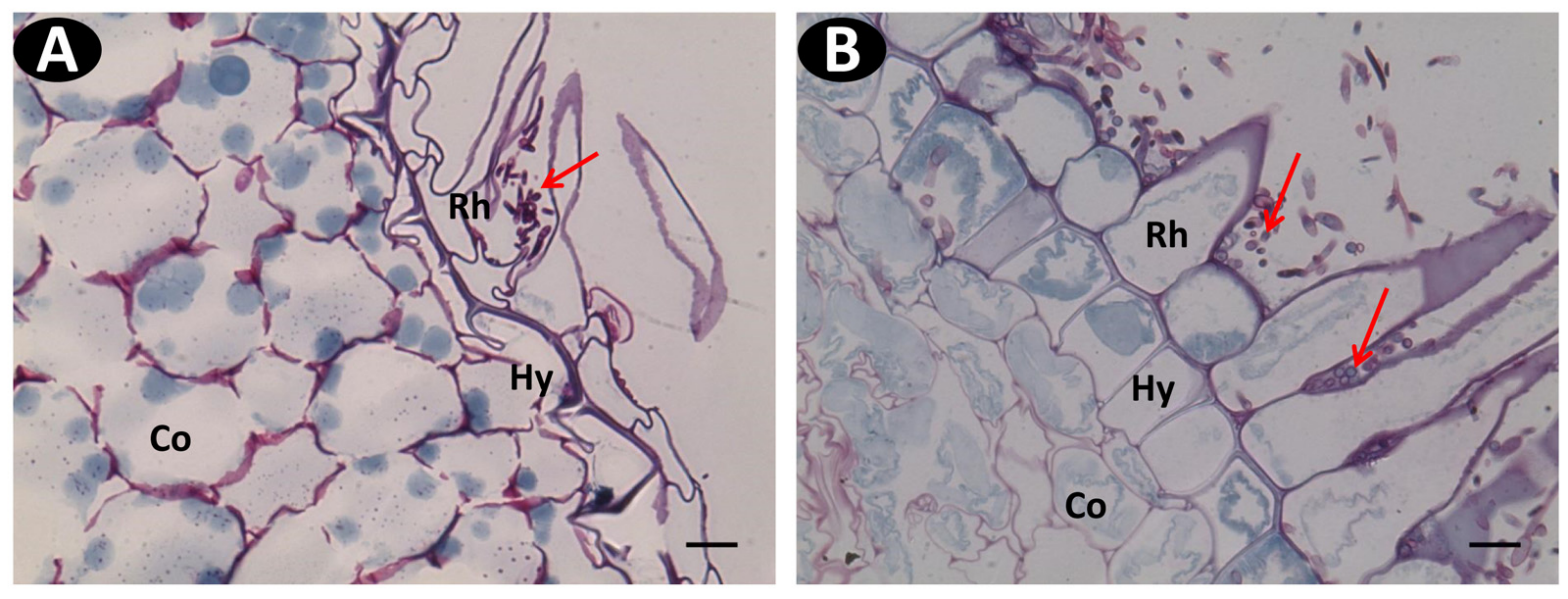
**Wide field microscopy images of transverse sections of Schiff Naphthol Blue-black double-stained vanilla root tissues infected with pathogenic Fo072 at 3 dpi. (A)** Transverse root section of the resistant accession pom0018 showing lesser colonization of hyphae (red arrows) in the hairy root region, compared to **(B)** susceptible accessions pla0001 showing abundant fungal colonization. Rh: Rhizodermis, Hy: hypodermis, Co: cortex. Scale bar = 20 μm.

**FIGURE 7:**
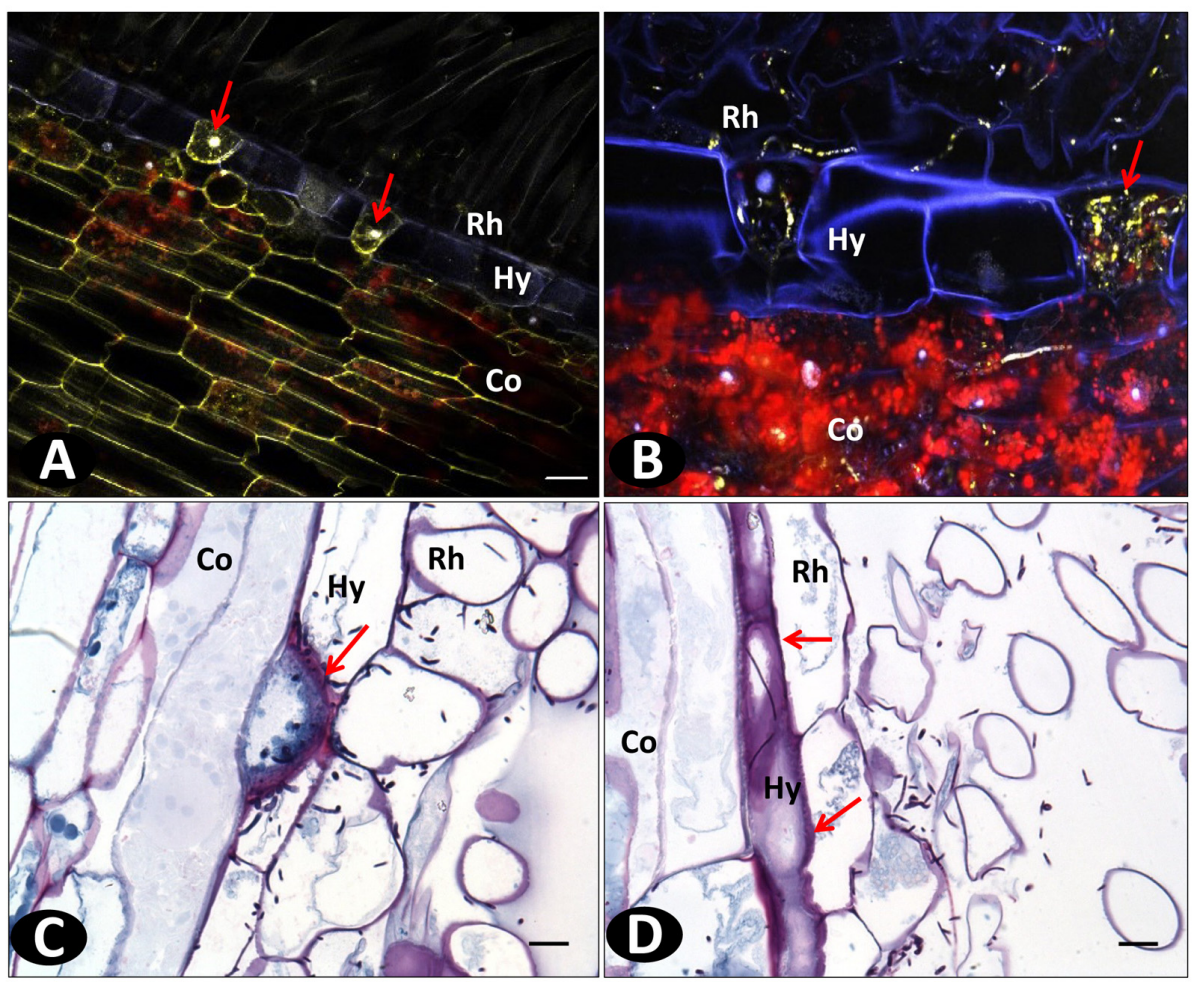
**Histopathological defense responses of resistant accessions infected with Fo072. (A,B)** Multiphoton images showing the formation of “globular” structures (pointed with arrows) on the hypodermal cells (Hy) entrapping the hyphae and preventing their further colonization to the cortical region (Co) of pom0018 infected with Fo072 at 4 dpi; **(C,D)** WFM images of infected root samples double-stained with Schiff reagent and Napthol Blue-black stain, showing the defense response of hypodermal cells (denoted with arrows) with **(C)** showing intense staining of hypodermal cell walls of pla0020 and **(D)** the formation of dark layered hypodermal cells between the rhizodermis (Rh) and cortex (Co) in pom0018 infected with Fo072 at 8 dpi. The pink color dark staining reveals the deposition of polysaccharides in the cell walls of hypodermis. Scale bar = 20 μm.

On resistant accessions pla0020 and pom0018, conidia germination and hyphae development was reduced (**Figure 6A**) compared to pla00001 (**Figure 6B**). A few hyphae reached the rhizodermis but were restricted to the hypodermal cells. In pom0018, these colonized cells were modified into globular structures that entrapped the hyphae (**Figures 7A–C**). In both resistant accessions (pla0020 and pom0018) an increased staining of the hypodermal cell wall was observed (**Figure 7D**) suggesting the formation of a polysaccharide layer in response to fungus penetration. No such modifications were observed in pla0001.

### Anatomical Differences between Susceptible and Resistant Cultivars

In order to further investigate the role of anatomical structures in plant defense, peripheral layer staining (FASGA, phloroglucinol and berberine hemisulfate) was carried out on uninfected roots using WFM.

**FIGURE 8:**
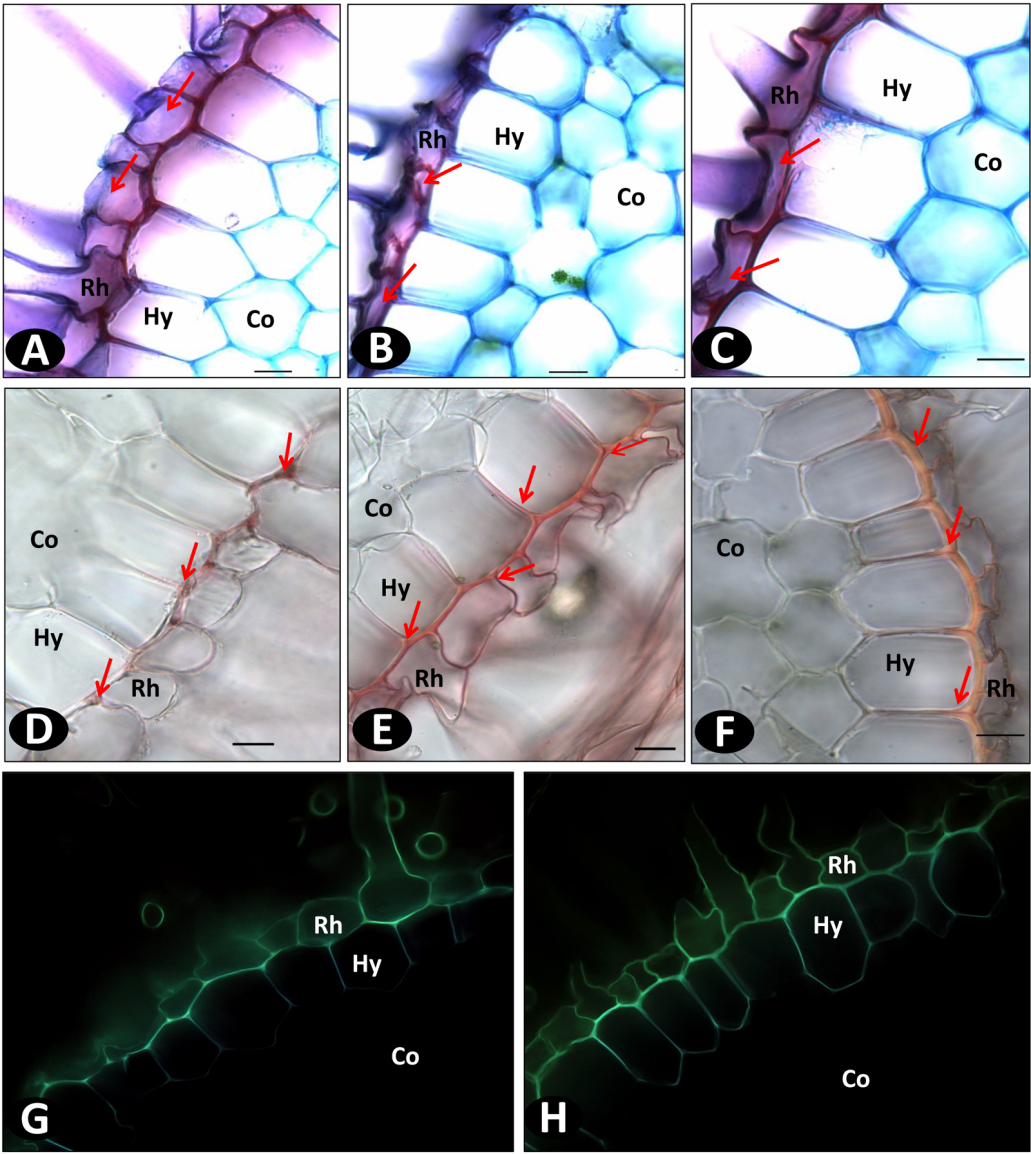
**Wide field microscopy images of non-inoculated *in vitro* roots showing the difference in the cell wall structure and compositions using different staining methods. (A–C)** FASGA stained transverse section of roots showing the round shaped rhizodermal (Rh) cells (denoted with arrows) in pla0001 **(A)** and compact rhizodermal cells in pla0020 **(B)** and pom0018 **(C)**; **(D–F)** Transverse sections of vanilla *in vitro* roots stained with phloroglucinol. Discontinuous lignin deposition mostly localized as small patches (denoted with arrows), without radial thickenings, on the hypodermis (Hy) of pla0001 **(D)**, contrary to pla0020 **(E)**, and pom0018 **(F)** showing continuous longitudinal and radial deposition of lignin on the longitudinal and radial walls of hypodermis. **(G,H)** displays the differences between susceptible pla001 **(G)** and resistant pom0018 **(H)** in the composition of rhizodermal and hypodermal cells stained with berberine hemisulfate which has affinity to suberin and lignin. Co: Cortex. Scale bar = 20 μm.

The rhizodermis of all genotypes is unilayered, with single celled root hairs, a common feature in most monocots. The cells forming the rhizodermis varied in shape between the three accessions. They were rounded and bigger in the susceptible pla0001 compared to the resistant pla0020 and pom0018 in which they were flattened and thinner (**Figures 8A–C**). However, in an *in vitro* comparison of a subset of 21 accessions ranging from resistant to HS, no significant correlation was observed between the size of epidermal cells and the resistance level of the accessions evaluated by AUDPC (*r* = 0.24, *P* = 0.293).

The hypodermis layer comprised uniseriate polygonal cells containing lignin thickenings on the outer longitudinal walls as shown by phloroglucinol staining (**Figures 8D–F**). However, the lignin staining revealed that the accessions had different types of hypodermis cell walls. In susceptible accessions (pla0001), the presence of lignin was discontinuous or very thin, while resistant accessions (pom0018 and pla0020) showed a strong and continuous lignin thickening on the outer walls of hypodermis. In addition, a radial thickening was present in resistant accessions but not in susceptible genotype (pla0001; **Figures 8E,F**). Phloroglucinol staining confirmed the higher lignin content on the hypodermal cell walls of resistant compared to susceptible accessions (**Figures 8G,H**).

The thickness of lignin deposition and presence of radial thickening was assessed on the same subset of 21 accessions of differing *Forv* resistance. A significant correlation coefficient was calculated between the hypodermal cell wall thickness and the mean AUDPC values (*r* = -0.72, *P* = 0.00024), and the presence of radial thickening was significantly related to AUDPC classes (*P* = 0.00012 in Fisher’s exact test for count data). High lignin thickening on the outer wall was consistently associated with radial thickening in all resistant accessions, except for cha0666 which was evaluated as moderately resistant (AUDPC = 10).

Lignin deposition was also observed in field-grown susceptible accession pla0001 and resistant accessions pla0020 and bah0086 using phloroglucinol staining. The results were consistent with *in vitro* observations (Supplementary Figure S5).

### Differential Spectral Response of Vanilla Accessions Inoculated with Fo072

**FIGURE 9:**
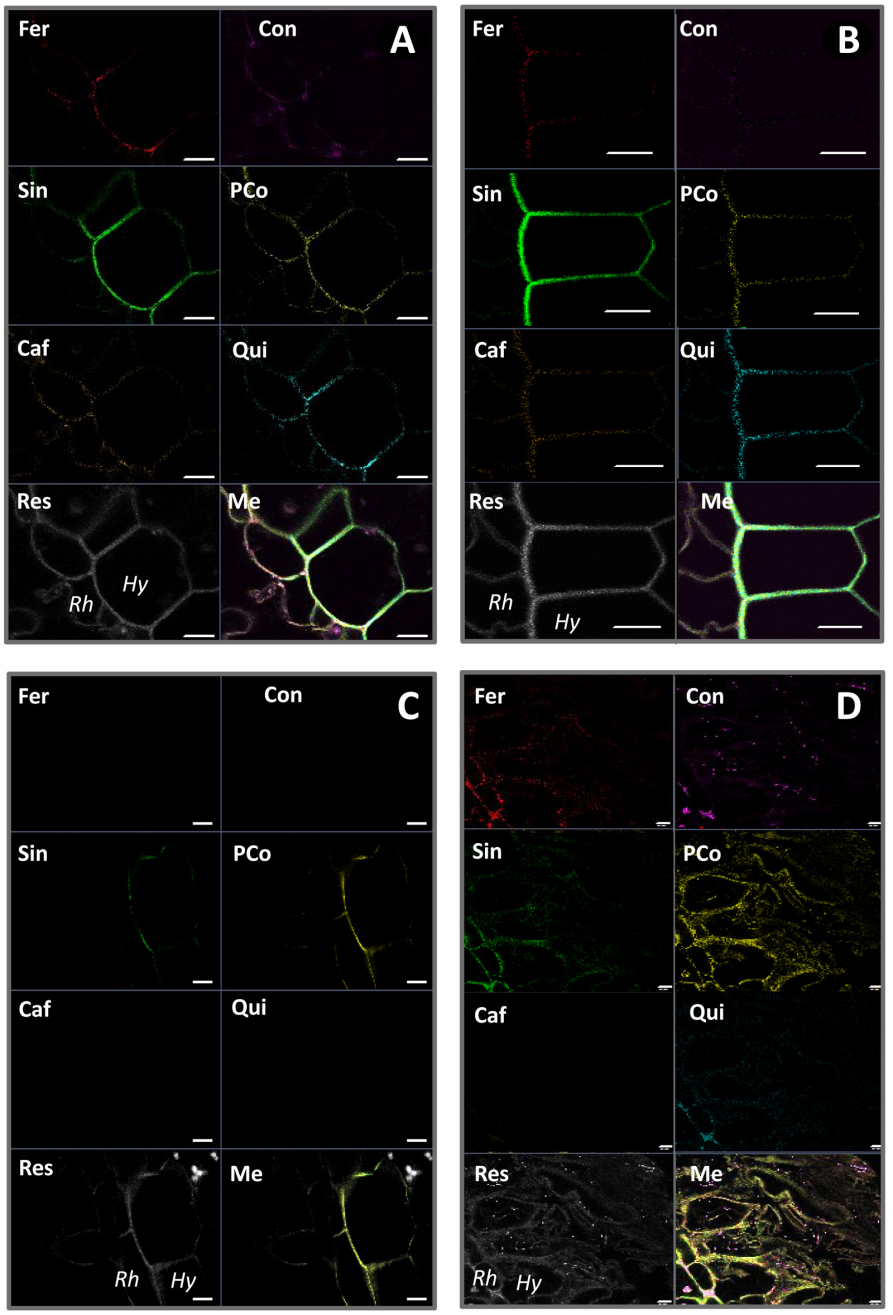
**Spectral unmixing analyses of non-inoculated roots of pla0001 (A) and pom0018 (B), and Fo072 inoculated root sections of pla0001 (C) and pom0018 (D) accessions, using six reference emission spectra compounds : Fer = ferulic acid; Con = conyferylic acid; Sin = sinapinic acid; pCo = p-coumaric acid; Caf = caffeic acid; Qui = quinic acid.** Res = residual channel and Me = merged image. Rh: rhizodermis, Hy: hypodermis. Scale bar: 20 μm.

Spectral analyses of the rhizodermal and hypodermal layers of the roots of susceptible (pla0001) and resistant (pom0018) accessions, both Fo72 inoculated and controls, were compared with reference spectra of four lignin precursors (ferulic acid, conyferylic acid, sinapinic acid, p-coumaric acid) and two others phenolic compounds (caffeic acid and quinic acid). On non-inoculated roots similar images were obtained for the six channels except conyferylic acid fluorescence which was slightly more continuous in the susceptible accession compared to the resistant (**Figures 9A,B**).

On the other hand, infected root samples of susceptible and resistant accessions displayed very divergent unmixed spectra. In the susceptible accession, the majority of the precursors were no longer present at 7 dpi, except sinapinic and p-coumaric acids which remained slightly visible on the hypodermis (**Figures 9A,C**). For the resistant accession, the p-coumaric and conyferylic acid impregnations were notably enhanced in the rhizodermal and hypodermal layers as well as in the cortex (**Figures 9B,D**), and not only in the walls but also in the cells for sinapic and p-coumaric acids.

## Discussion

Root and stem rot is a major challenge for vanilla cultivation worldwide. The lack of knowledge about *Forv*-vanilla interactions and the limited genetic resources available have hampered the development of resistant varieties which is acknowledged as the best means for controlling *Fusarium* diseases. In this study, using BRC Vatel’s diverse collection of vanilla accessions and powerful image acquisition and analysis technology we thoroughly documented three aspects of host-pathogen interactions. First of all, the penetration and invasion route of the fungus into the plant was precisely described as well as subsequent plant responses. Secondly, several anatomical structures involved in RSR resistance were unveiled in two *Vanilla* accessions including, for the first time, *V. planifolia*. Finally, more than one hundred vanilla accessions were assessed for *Forv* resistance, and a simple, quick and reliable assay was developed which will enhance *Forv* resistance evaluation of novel genetic resources.

### *Forv* Infection and Colonization Pattern

To date, there is only a single published work describing the penetration process and colonization pattern of a fungus in vanilla plants (Alconero, 1968). Our histological studies outlined the route and time frames of *Fusarium* penetration and colonization in vanilla roots. In our experimental conditions, the germination of conidia and subsequent infection was triggered as early as 2 dpi. Similarly to *F. oxysporum* f. sp. *radicis-cucumerinum* and *F. oxysporum* f. sp. *radicis lycopersici* (Lagopodi et al., 2002; Cohen et al., 2014) the primary infection site of *Forv* was the hairy root region and the fungus penetrated directly through the rhizodermis in the absence of cell wounds. This was in disagreement with previous report from Alconero (1968) who described the penetration of *Fusarium* through the root apex of vanilla plants and concluded from field and laboratory observations that penetration was mainly through wounds caused by insects and nematodes. The hypodermis passage cells regulate the apoplastic entry of water and other solutes to the cortex. These passage cells also act as the entry point for fungi (pathogenic or mycorrhiza) to access the cortical region of the root (Esnault et al., 1994; Kamula et al., 1994; Sharda and Koide, 2008; Chomicki et al., 2014). This was confirmed in the case of *Forv* in vanilla where the pathogen always entered the cortex through the passage cells as observed by Alconero (1968).

Contrary to vascular *formae speciales* of *F. oxysporum* which cause sudden wilt of plants by rapidly invading the vascular bundles (Li et al., 2011; Ndambi et al., 2012; Araujo et al., 2014) *Forv* had not reached the root’s vascular system by 9 dpi indicating that the virulence of *Forv* was mainly due to the destruction of cortical cells of the hairy area of the root. The infected root is therefore capable of reemitting a functional root upstream of the infected area of the root, thus enabling root regeneration and plant survival for months in the field. Our study focused on root infections. However, *Forv* also infects stem tissues in the field (Pinaria et al., 2010). In a few instances we observed fungal penetration in the stem occurring at the root-stem junction after external growth of hyphae along aerial roots (data not shown). This second entry point needs to be better described in order to study further the RSR symptomatology and epidemiology.

### Cellular and Histochemical Mechanisms in *Forv* Resistance

In resistant accessions pla0020 and pom0018, penetration occurred less frequently than in the susceptible accession, and invasion was mostly limited to the hypodermal cells. This minor infection caused no apparent damage to the root system, leaving the plant unharmed. Morphological, histochemical, and physiological variations in the hypodermal cells could contribute to the resistance of these accessions.

First, in the non-inoculated plants, the neat correlations between hypodermal cell wall thickness and AUDPC scores supported the hypothesis that hypodermal lignin constitutively deposited on cell wall contributes to resistance of vanilla to *Forv*. The hypodermal wall thickness of resistant accessions was thicker (5 μm) than in susceptible accessions (1–2 μm). The visualization of lignin by phloroglucinol staining and of lignin precursors by spectral analysis confirms the continuity of the hypodermal wall on all sides of the cell in resistant genotypes. It is assumed that lignified cell walls are less susceptible to degradation than cellulose cell walls thereby hampering hyphal penetration and colonization of the intercellular spaces. The role of sub-epidermal cell thickenings in the prevention of *Pseudocercosporella* attack have been described in wheat (Murray and Bruehl, 1983). It is possible nevertheless that this constitutive feature preventing the intercellular movement of fungus by peripheral and radial lignification of cell walls could be enhanced by the pathogen as suggested by images of root sections from field plants.

Second, the accumulation of phenolic compounds forming a barrier in the hypodermal region and thereby preventing the pathogen from colonizing the cortical region have been reported in many plants (Brammall and Higgins, 1988; Tessier et al., 1990; Olivain and Alabouvette, 1997; Fang et al., 2012). Such a mechanism is likely to occur in vanilla as evidenced by histochemical root studies that showed thickening specifically in the hypodermal cell wall of inoculated resistant genotypes. Results of spectral analysis showed differences in peripheral cell wall composition between pre- and post-infection stages. The higher concentration of sinapic acid in resistant genotypes, closely related to phloroglucinol stain, suggest the important role of S-lignin in these walls. An increase in the level of p-coumaric and conyferilic acid in resistant genotypes were observed after pathogen infection. Similar mechanisms were observed in many hosts, where the *Fusarium* elicitors enhance the accumulation of phenolic compounds like ferulic, p-coumaric, caffeic, salicylic, sinapic, and vanillic acids in cell walls (de Ascensao and Dubery, 2003; Mandal and Mitra, 2007; Voxeur et al., 2015). The esterification of these compounds in the cell walls forms a physical barrier against fungal penetration and enzymes responsible for damaging cell walls. Thus the increased level of lignin precursors may be implicated in the resistance responses of vanilla to *Forv* infection (Hammerschmidt and Kuc, 1982; Cvikrová et al., 1993; El Modafar and El Boustani, 2001; de Ascensao and Dubery, 2003; Zeitoun et al., 2014).

Third, specific cellular entrapment of hyphae on the hypodermis was noted on resistant accession pom0018, suggesting a defensive role of hypodermal cells. Cellular reactions in the hypodermis with the formation of wall inclusions and appearance of sensitive cells in response to the hyphae have been previously reported (Beswetherick and Bishop, 1993; Olivain and Alabouvette, 1999). The entrapment of hyphae in the hypodermis serves as a barrier, limiting the extension of hyphae to the cortical region. This phenomenon has not been observed in resistant *V. planifolia* pla0020 and might be specific of *V. pompona*.

Finally, conidial germination and hyphae development were notably greater on the roots of susceptible accessions compared to the resistant accession. This could be related to the high content of antifungal phenolic compounds like coumaric, cinnamic, ferulic, salicylic, and sinamic acids evidenced in resistant roots by spectral analysis. Indeed, the role of phenolic root exudates in limiting conidial germination was demonstrated *in vitro* for a number of plant species (Steinkellner et al., 2005; Wu et al., 2008; Liu et al., 2009; Singh et al., 2010; Ling et al., 2013). As a whole several mechanisms were identified that could reduce root infection of resistant vanilla plants. The multiplicity of these mechanisms could account for the graduated levels of resistance found in the vanilla germplasm evaluated in this study.

### Screening for Resistance to *Forv* in Vanilla

An unprecedented range of *Vanilla* genotypes has been assessed for RSR resistance in long term assays in the field and in controlled *in vitro* conditions. The 254 accessions tested belonged to 18 species and six hybrids.

There was a good congruency between field and *in vitro* evaluation results except for two accessions. This is the case for pla0038 which has been grown successfully in the field for more than 10 years despite being graded as very susceptible in *in vitro* assays. This discrepancy could result from the presence of favorable microflora in the compost providing protection of the root system, as shown in some soils (Alabouvette et al., 2009). Nonetheless, it seems very unlikely that this protective compost occurred in only three adjacent locations of the shadehouse were pla0038 was cultivated. Another possibility is that pla0038 harbors some kind of protective endophyte (Ordonez et al., 2013; White et al., 2014; Khoyratty et al., 2015) and that this protective endophyte had been eliminated from *in vitro* plants during tissue culture. Checking lignin impregnation of hypodermal cell walls as well as field trial in different soil conditions could bring additional information about the status of this questionable accession.

In the first rating of field plants using seven growth and symptoms variables, a horseshoe distribution of individuals (Guttman effect) was observed in the first factorial plane of MCA (**Figure 1**). This may result from the fact that resistant genotypes as well as the very susceptible accessions both produced a reduced number of roots compared to MS accessions. Roots remained functional in resistant accessions while in very susceptible accessions the disease strongly limited growth and ability to produce new roots. Conversely, the MS accessions had slightly reduced growth and were capable of producing numerous new roots in response to root rot.

Root and stem rot resistance assays confirmed the high resistance level of *V. pompona* as well as the resistance of eight hybrids of *V. planifolia* with *V. pompona* and *V. phaeanta* (Tucker, 1927; Tonnier, 1960; Delassus, 1963). They also revealed novel sources of RSR resistance such as *V. bahiana*, a close relative to the resistant species *V. phaeanta, V. sp. CR0068* and *V. crenulata*. Most of the *V. planifolia* accessions, *V. × tahitensis* and *V. odorata* were susceptible to RSR. But interestingly, seven accessions (including pla0020) obtained from autopollination of an ordinary *V. planifolia* revealed a high level of resistance to Fo072. This intraspecific diversity in *Forv* resistance might result from the high level of heterozygosity of *V. planifolia* (Soto Arenas, 1999; Bory et al., 2008b). Moreover, the fact that most *V. planifolia* accessions are susceptible to *Forv* and only seven out of the 48 selfed-progenies of *V. planifolia* plants tested resistant to *Forv*, may suggest that the resistance to *Forv* in *V. planifolia* could be governed by a major recessive factor. This finding therefore demonstrated that a breeding strategy based on *V. planifolia* or *V. × tahitensis* selfing could be efficient for obtaining progenies gathering both resistance to *Forv* and true to type aromatic profile of the fruits. Indeed, this last characteristic is generally lost in interspecific hybrids (Theis and Jimenez, 1957; Belanger and Havkin-Frenkel, 2010). The strategy using selfed-progenies, combined with the efficient *in vitro Forv*-resistance assay developed in this study could help create new vanilla varieties much needed by the vanilla industry. Furthermore, the knowledge about histological location and temporality of vanilla responses to *Forv* infection opens avenues for investigating the genetics and the mechanisms at work in the RSR resistant vanilla genotypes.

## Author Contributions

SK, GC, JV, PB, and MG, conceived and designed the experiments.

SK, GC, FM-L, FG, JD, KJ, and MG, performed the experiments.

SK, GC, JV, FC, PB, and MG, analyzed the data.

SK, GC, JV, FC, PB, and MG, wrote the article.

## Conflict of Interest Statement

The authors declare that the research was conducted in the absence of any commercial or financial relationships that could be construed as a potential conflict of interest.

## Supplementary material

FIGURE S1 **Two *V. planifolia* accessions showing contrasted development into shade house 4 years after planting.** On the left, pla0670 shows reduced vegetative growth, decaying stem and leaves and abundant descending roots that die on ground; on the right pla0695 shows good growth and limited root symptoms.

FIGURE S2 **Scatterplots of first factorial plane of MCA for 128 vanilla accessions rated for RSR symptoms in the field using seven variables of four to five levels (see **Table 2**): PS: plant size, SN : number of looped stems, SD: percentage of grooved stems and decaying leaves, PV: vigor of the plant (size and number of new shoots), NR: number of roots descending along the stalk, DR: proportion of dried roots among the descending roots, AR: number of aerial roots**.

FIGURE S3 **Correspondence between RSR rating in 2009, represented by plant size (PS) class (see **Table 2**) and the RSR level rated in 2013, for 125 vanilla accessions grown under shadehouses**.

FIGURE S4 **Mycelium development and rot on *in-vitro* plantlets (pla0001) inoculated with Fo072 at: **(A)** 0 dpi, **(B)** 5 dpi, **(C)** 12 dpi, and **(D)** mock-inoculated *in-vitro* plantlets**.

FIGURE S5 **Wide field microscopy (WFM) images of the transverse root sections of field grown plants stained with phloroglucinol, showing distinct composition of lignified hypodermal (Hy) cell walls of pla0001 **(A)**, with discontinuous lignin, seen as patches on the outer longitudinal wall (marked with arrows), compared to pla0020 **(B)** and bah0086 **(C)** showing the continuous layer of lignin present on the longitudinal walls and the presence of inner radial walls on the hypodermis**.

TABLE S1 **Correspondence between *in vitro* rating based on AUDPC and RSR-levels rated in the field in 2013 for 26 accessions rated with two methods. HS highly susceptible, S susceptible, MS moderately susceptible, SR slightly resistant, R resistant**.
