# Supplementary figures and images for: Differential Responses of Vanilla Accessions to Root Rot and Colonization by Fusarium oxysporum f. sp. radicis-vanillae
Source: Front Plant Sci. 2015 Dec 18;6:1125. doi: 10.3389/fpls.2015.01125 (PMC4683197; doi:10.3389/fpls.2015.01125)

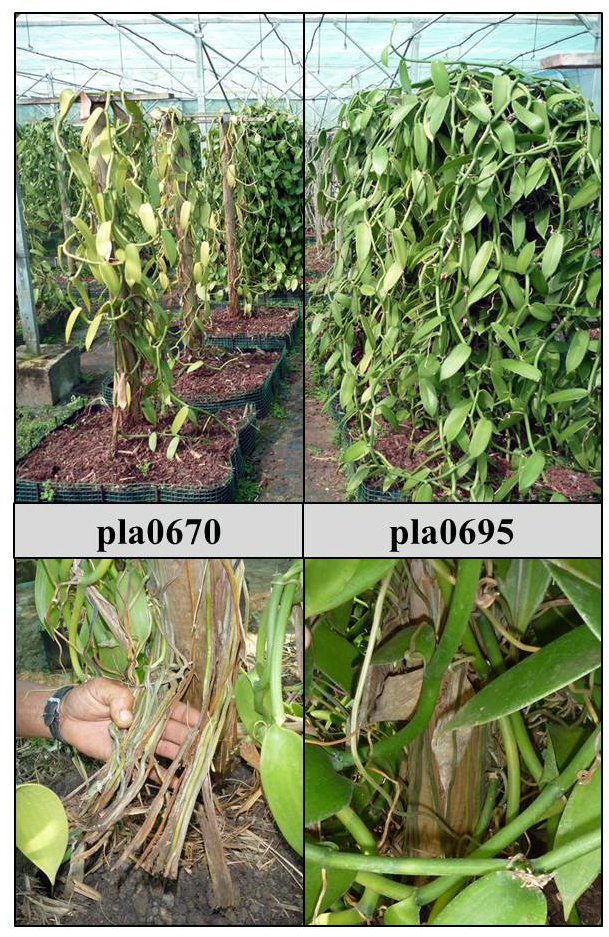

Supplement: FIGURE S1 — Two V. planifolia accessions showing contrasted development into shade house 4 years after planting. On the left, pla0670 shows reduced vegetative growth, decaying stem and leaves and abundant descending roots that die on ground; on the right pla0695 shows good growth and limited root symptoms. [file Image_1.JPEG]

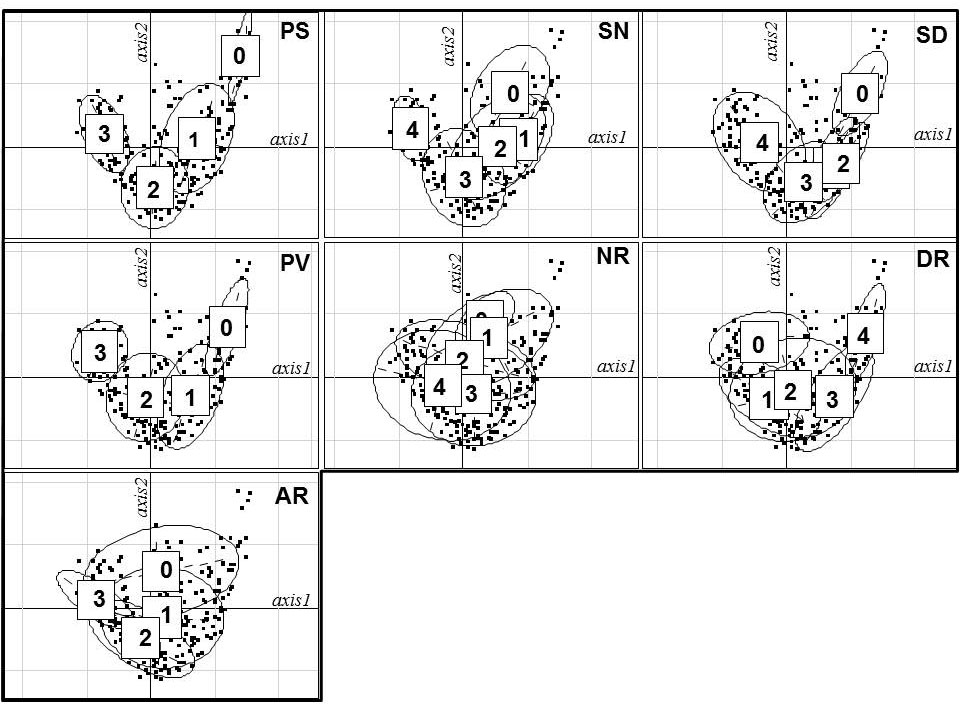

Supplement: FIGURE S2 — Scatterplots of first factorial plane of MCA for 128 vanilla accessions rated for RSR symptoms in the field using seven variables of four to five levels (see Table 2): PS: plant size, SN : number of looped stems, SD: percentage of grooved stems and decaying leaves, PV: vigor of the plant (size and number of new shoots), NR: number of roots descending along the stalk, DR: proportion of dried roots among the descending roots, AR: number of aerial roots. [file Image_2.JPG]

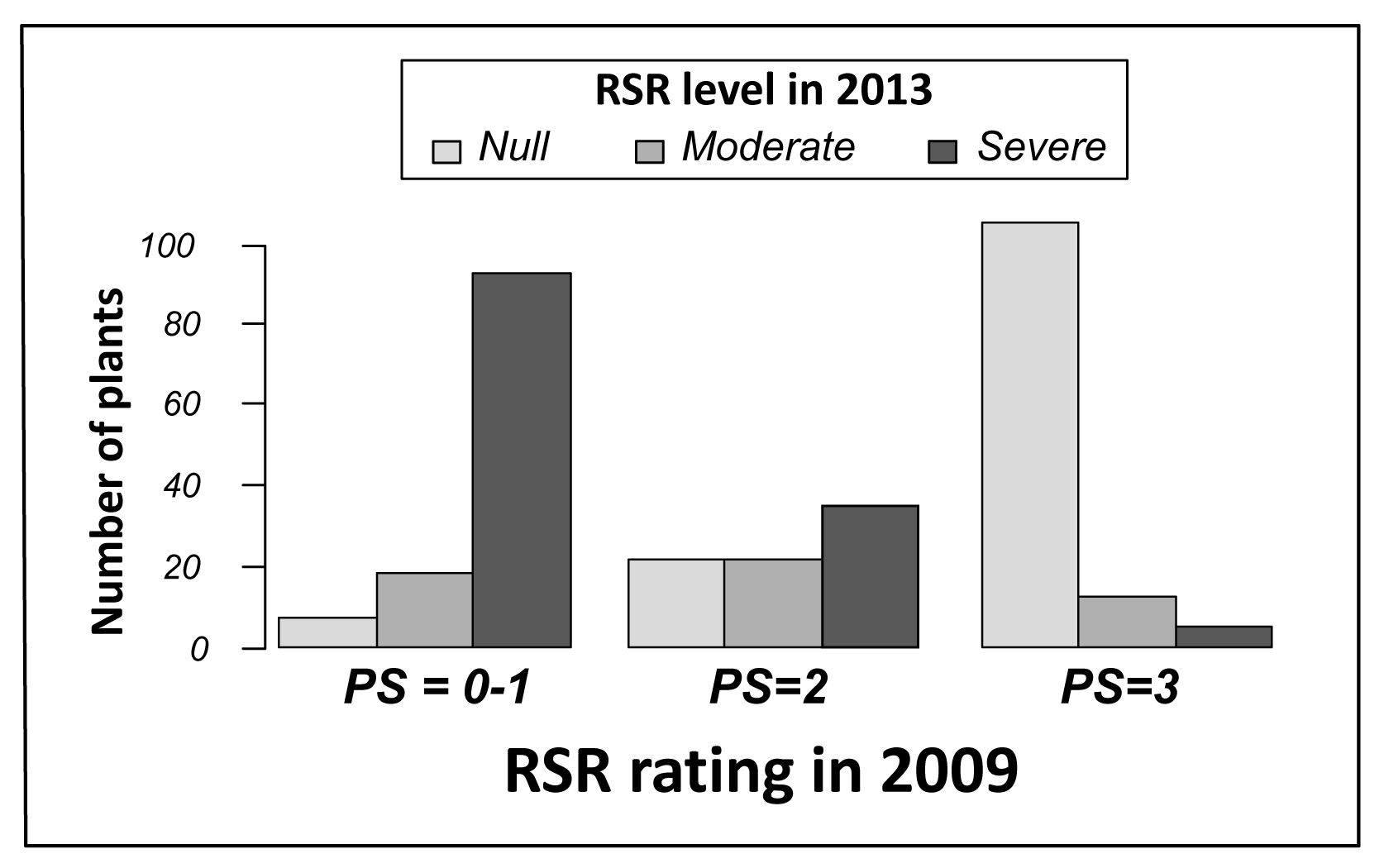

Supplement: FIGURE S3 — Correspondence between RSR rating in 2009, represented by plant size (PS) class (see Table 2) and the RSR level rated in 2013, for 125 vanilla accessions grown under shadehouses. [file Image_3.JPEG]

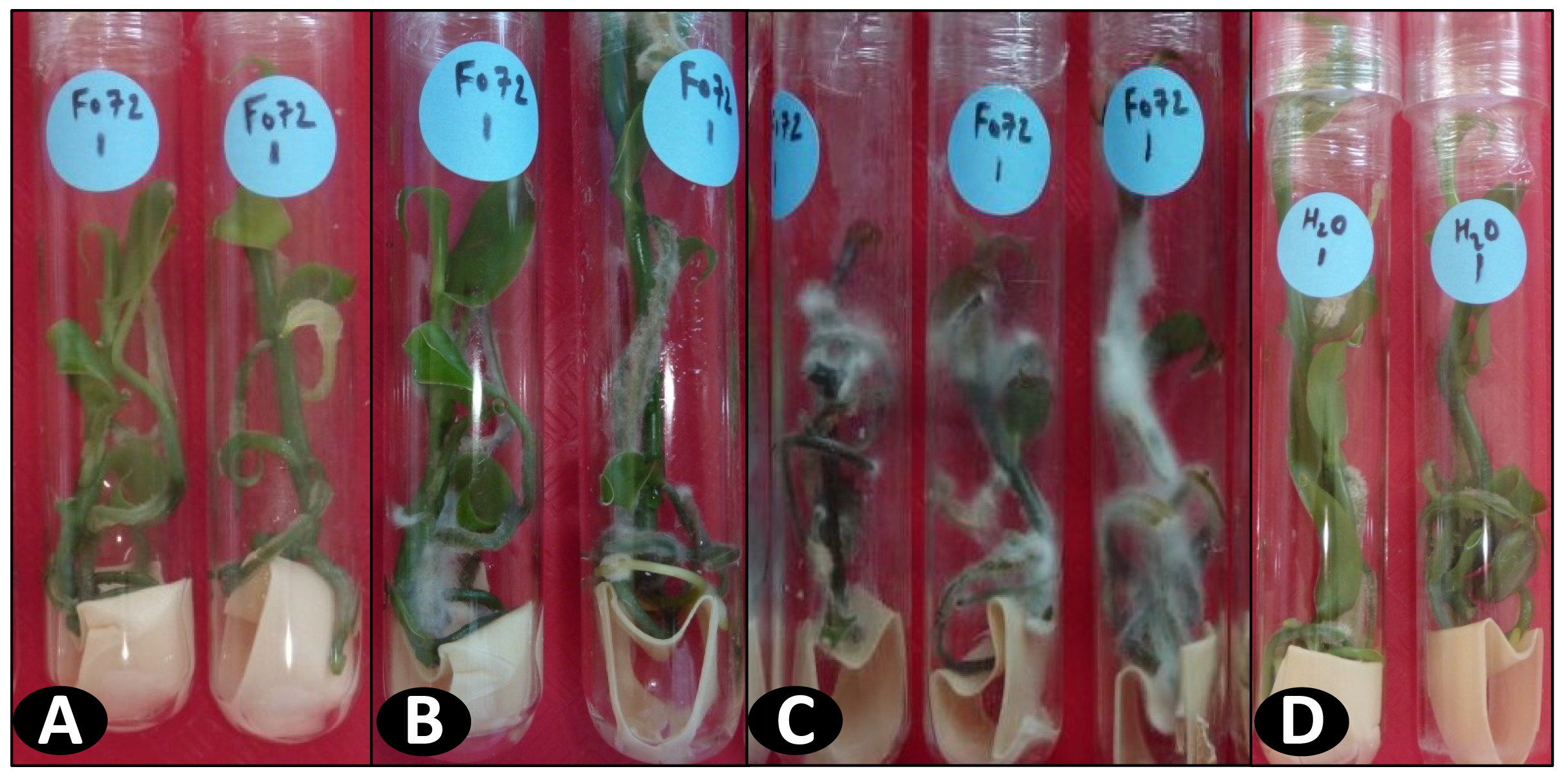

Supplement: FIGURE S4 — Mycelium development and rot on in-vitro plantlets (pla0001) inoculated with Fo072 at: (A) 0 dpi, (B) 5 dpi, (C) 12 dpi, and (D) mock-inoculated in-vitro plantlets. [file Image_4.JPEG]

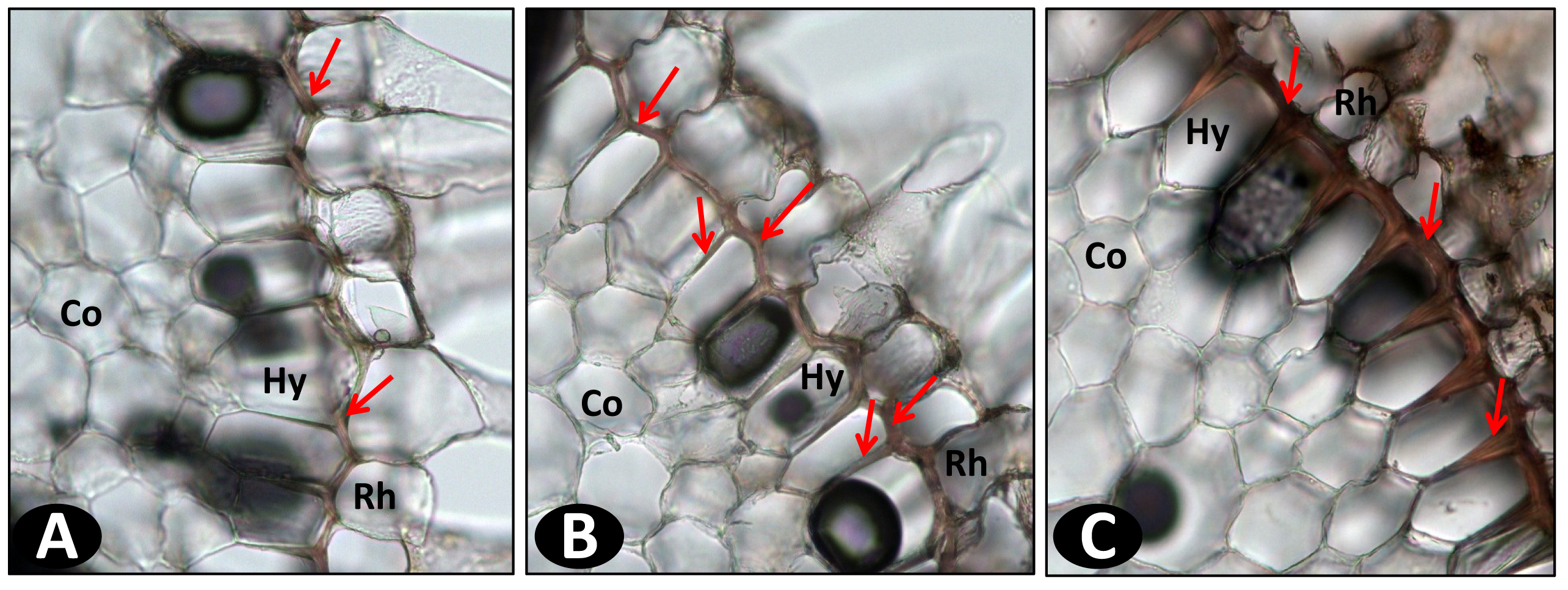

Supplement: FIGURE S5 — Wide field microscopy (WFM) images of the transverse root sections of field grown plants stained with phloroglucinol, showing distinct composition of lignified hypodermal (Hy) cell walls of pla0001 (A), with discontinuous lignin, seen as patches on the outer longitudinal wall (marked with arrows), compared to pla0020 (B) and bah0086 (C) showing the continuous layer of lignin present on the longitudinal walls and the presence of inner radial walls on the hypodermis. [file Image_5.JPEG]
